# Cost-Effectiveness of Ipilimumab Plus Anti-PD-1 Therapy *Versus* Ipilimumab Alone in Patients With Metastatic Melanoma Resistant to Anti-PD-(L)1 Monotherapy

**DOI:** 10.3389/fonc.2021.743765

**Published:** 2021-11-09

**Authors:** Ye Peng, Xiaohui Zeng, Liubao Peng, Qiao Liu, Lidan Yi, Xia Luo, Sini Li, Liting Wang, Shuxia Qin, Xiaomin Wan, Chongqing Tan

**Affiliations:** ^1^ Department of Pharmacy, The Second Xiangya Hospital of Central South University, Changsha, China; ^2^ PET-CT Center, The Second Xiangya Hospital of Central South University, Changsha, China

**Keywords:** ipilimumab plus anti-PD-1, retrospective analysis, cost-effectiveness, melanoma, Markov model

## Abstract

**Objective:**

The use of ipilimumab plus anti-PD-1 has recently been shown to significantly improve the survival of patients with metastatic melanoma resistant to anti-PD-(L)1 monotherapy. The study assessed the cost-effectiveness of ipilimumab plus anti-PD-1 therapy in this population from the US payer perspective.

**Materials and Methods:**

A Markov model was created based on a retrospective analysis of patients with metastatic melanoma who were resistant to anti-PD-(L)1. Cost information was obtained from the Centers for Medicare and Medicaid Services and literature-based costs. The utility value was derived from the published literature. The results of the model was the total cost, quality-adjusted life-year (QALY), and incremental cost-effectiveness ratio (ICER). The uncertainty of the model was addressed through sensitivity analysis. In addition, we also conducted subgroup analysis.

**Results:**

Ipilimumab plus anti-PD-1 provided an improvement of 1.39 QALYs and 2.48 LYs, at a ICER of $73,163 per QALY. The HR of OS was the variable that had the greatest impact on ICER. Compared to ipilimumab, the probability of ipilimumab plus anti-PD-1 being cost-effective was 94% at the WTP of $150,000/QALY. The results of the subgroup analysis showed that the ICER in the majority of the subgroups was less than $150,000/QALY.

**Conclusions:**

Ipilimumab plus anti-PD-1 was likely to be cost-effective compared to ipilimumab for patients with metastatic melanoma who are resistant to anti-PD-(L)1 at a WTP threshold of 150,000/QALY.

## Introduction

Skin cancer is a commonly diagnosed cancer in the United States, and more than 2 million people are diagnosed annually (1). It is estimated that there will be 106,110 new cases of skin melanoma and 7,180 deaths in the United States in 2021 (2). And the 5-year survival rate for patients with stage IV melanoma is only 19% (3).

At present, immune checkpoint inhibitors have revolutionized the treatment of advanced melanoma (4). However, the majority of patients are resistant to anti-PD-1 therapy and require further treatment (4). Recently, a retrospective analysis by Pires da Silva et al. showed that ipilimumab plus anti-PD-1 (pembrolizumab or nivolumab) has higher efficacy than ipilimumab in patients with resistance to anti-PD-(L)1 (4). The median overall survival (OS) of the ipilimumab plus anti-PD-1 group was 24.0 months, which was 11.6 months longer than that of ipilimumab. The proportion of patients with grade ≥ 3 adverse events (AEs) was similar in the two groups.

Considering the limited number of randomized clinical trials and the uncertainty of their external validity, cost-effectiveness analysis based on real-world data can provide clinicians and decision-makers with more valuable information. In daily clinical practice, the effect of ipilimumab plus anti-PD-1 as a second-line treatment for patients with metastatic melanoma on its health outcomes and costs remained unclear. Therefore, this study evaluated the cost-effectiveness of ipilimumab plus anti-PD-1 therapy in the treatment of patients with metastatic melanoma resistant to anti-PD-(L)1 monotherapy from a US payer perspective.

## Materials and Methods

### Study Design

**Figure 1:**
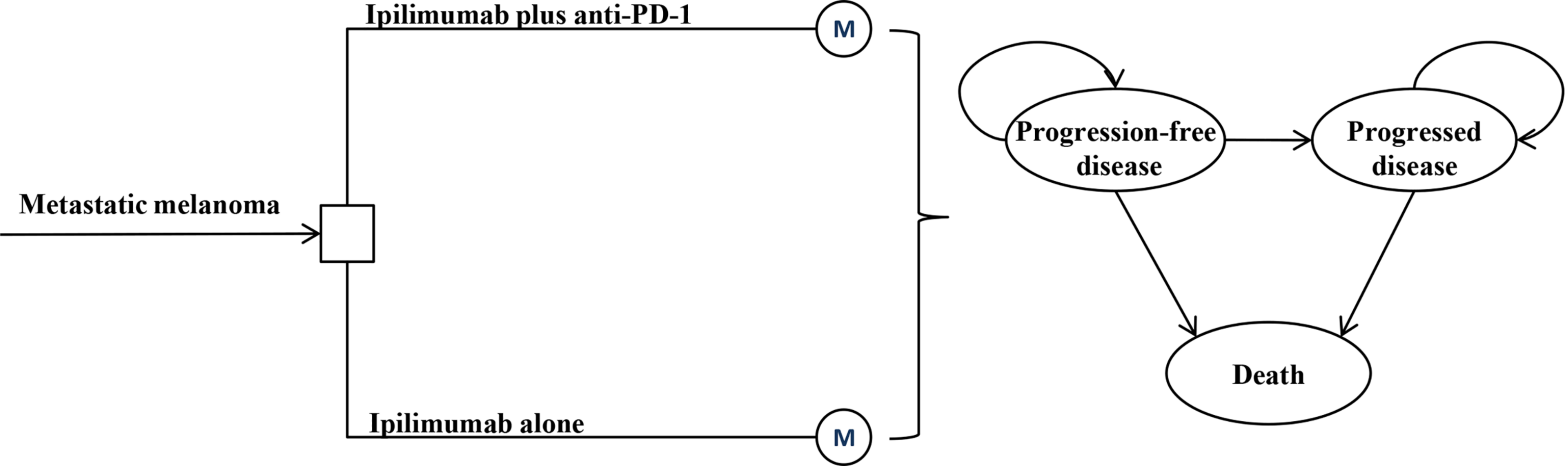
Model structure for metastatic melanoma resistant to anti-PD-(L)1 monotherapy.

A Markov model was constructed to evaluate the lifetime cost and outcome of ipilimumab plus anti-PD-1 therapy for metastatic melanoma with anti-PD-(L)1 resistance (**Figure 1**). ALL patients initially entered the model and received either ipilimumab monotherapy or ipilimumab plus anti-PD-1 (4). Once the disease progressed, patients received the best supportive care (BSC) until death. A three-week Markov cycle and a 3% annual discount rate were used to estimate the costs and outcomes associated with the two treatment strategies (5). The time horizon of this analysis was the lifetime. The results of the model was the total cost in 2021 USD, quality-adjusted life-year (QALY), and incremental cost-effectiveness ratio (ICER). A willingness-to-pay (WTP) threshold of $150,000/QALY was applied for results analysis (6). TreeAge Pro (TreeAge Software, Williamstown, MA) and R were used for statistical analysis.

### Clinical Inputs

The base-case estimation of transition probabilities was shown in **Table 1**. The transition probability of disease progression in the ipilimumab arm was derived from the study of Pires da Silva et al. using standard extrapolation techniques (4, 7). First, Individual patient-level data of the ipilimumab arm were reconstructed based on the progression-free survival (PFS) and OS Kaplan-Meier curve. Then, these reconstructed survival data were used to fit the following parametric functions; Exponential, Weibull, Lognormal, Log-logistic, Gompertz, and Generalized gamma. Finally, the appropriate parametric distribution was selected based on statistical measures of the Akaike information criterion and visual inspection. The progression rate of ipilimumab plus anti-PD-1 arm was calculated based on the hazard ratios (HRs) reported in the study by Pires da Silva et al (4).

**Table 1:** Model parameters.

Variable	Baseline value	Minimum	Maximum	Distribution
Log-logistic OS survival model with ipilimumab	Theta=0.03916796, Kappa=1.52458	–	–	–
Log-logistic PFS survival model with ipilimumab	Theta=0.1380415, Kappa=1.922389	–	–	–
HR of ipilimumab plus anti-PD-1 *vs* ipilimumab for OS	0.50 (4)	0.38	0.66	Lognormal
HR of ipilimumab plus anti-PD-1 *vs* ipilimumab for PFS	0.69 (4)	0.55	0.87	Lognormal
Percentage of achieving treatment response in ipilimumab plus anti-PD-1 group	0.31 (4)	0.25	0.37	Beta
Percentage of achieving treatment response in ipilimumab group	0.13 (4)	0.10	0.16	Beta
Utility				
Complete/partial response	0.88 (12)	0.70	1.00	Beta
SD	0.80 (12)	0.64	0.96	Beta
PD	0.52 (12)	0.42	0.62	Beta
Drug cost				
Ipilimumab per mg	166 (8)	133	199	Gamma
Nivolumab per mg	30 (8)	24	36	Gamma
Pembrolizumab per mg	55 (8)	44	66	Gamma
BSC	4,492 (9)	3,594	5,390	Gamma
Disease management in PFD state on treatment per week	189 (9)	151	227	Gamma
Disease management in PFD state off treatment per week	590 (9)	472	708	Gamma
Terminal care	18,042 (9)	14,434	21,650	Gamma
Administration cost per cycle				
First hr^a^	148 (10)	119	178	Gamma
Additional hr^b^	31 (10)	25	38	Gamma

OS, overall survival; PFS, progression-free survival; HR, hazard ratio; SD, stable disease; PD, progressed disease; BSC, best supportive care; PFD, progression-free disease.

a Administration cost for first hour chemotherapy infusion.

b Administration cost for additional hour chemotherapy infusion.

### Costs and Utilities

All cost information in the model was shown in **Table 1**. Based on the study of Pires da Silva et al. (4), 99% of the patients in the ipilimumab plus anti-PD-1 group received ipilimumab plus nivolumab and 1% of the patients received ipilimumab plus pembrolizumab.The cost of ipilimumab, nivolumab, and pembrolizumab was 106% of the average sales price (8). A body weight of 70 kg was used to calculate the dose administered (9). The dosing schedule was based on the prescribing information of the US Food and Drug Administration (FDA): ipilimumab at a dose of 3 mg/kg plus nivolumab at a dose of 1mg/kg every 3 weeks for 4 doses, followed by nivolumab at a dose of 240 mg every 2 weeks (ipilimumab plus nivolumab strategy); ipilimumab at a low-dose of 1 mg/kg plus pembrolizumab at a dose of 2mg/kg every 3 weeks for 4 doses, followed by pembrolizumab at a dose of 200 mg every 3 weeks (ipilimumab plus pembrolizumab strategy); and ipilimumab at a dose of 3 mg/kg every 3 weeks for 4 doses (ipilimumab strategy). Administration costs were derived from the 2021 Centers for Medicare & Medicaid Services Physician Fee Schedule (10). There was no significant difference in the incidence of grade 3-5 adverse events (AEs) between the ipilimumab arm and ipilimumab plus anti-PD-1 arm based on the study of Pires da Silva et al. (4) (33% *vs* 31%). Therefore, the cost and disutility of AEs were not included in the model. The overall costs of BSC and terminal care were based on previously published literature (9). All costs were adjusted to 2021 USD using the US consumer price index (11).

The utility value was derived from the published litersture (12). The utility values for stable disease, response, and progressed disease were 0.80, 0.88, and 0.52, respectively (12). Stable disease and response were sub-states of the progression-free disease state, which have different quality-of-life. QALYs were obtained by weighting patient survival according to utility value.

### Sensitivity Analysis

A series of sensitivity analyses were incorporated to assess uncertainty. In one-way sensitivity analyses, each model parameter was changed within the ranges listed in **Table 1** to explore their influence on the results. The range of all parameters was their 95% CIs or ±20% of the baseline value. In probabilistic sensitivity analysis, 10,000 Monte Carlo simulations were performed by setting a distribution for each parameter. Costs were described by gamma distributions, HRs by a lognormal distribution, and utilities and probabilities by beta distributions. Subgroup analysis was also performed in the pre-specified subgroups reported in the study of Pires da Silva et al.

## Results

### Base-Case Analysis

In comparison with the ipilimumab arm, ipilimumab plus anti-PD-1 provided an improvement of 1.39 QALYs and 2.48 life-years (LYs) (2.17 *vs* 0.79 QALYs and 3.80 *vs* 1.31 LYs, respectively). However, ipilimumab plus anti-PD-1 was also associated with a significantly higher cost ($243,480 *vs* $142,083), resulting in an ICER of $73,163 per QALY (**Table 2**).

**Table 2:** Base case results.

Results	Ipilimumab	Ipilimumab plus anti-PD-1	Incremental
LYs	1.31	3.8	2.48
QALYs	0.79	2.17	1.39
Total cost, $	142083	243480	101,397
ICER, $			
Per LY	–	–	40,820
Per QALY	–	–	73,163

LYs, life years; QALYs, quality-adjusted life-years; ICER, incremental cost-effectiveness ratio.

### Sensitivity Analysis

**Figure 2:**
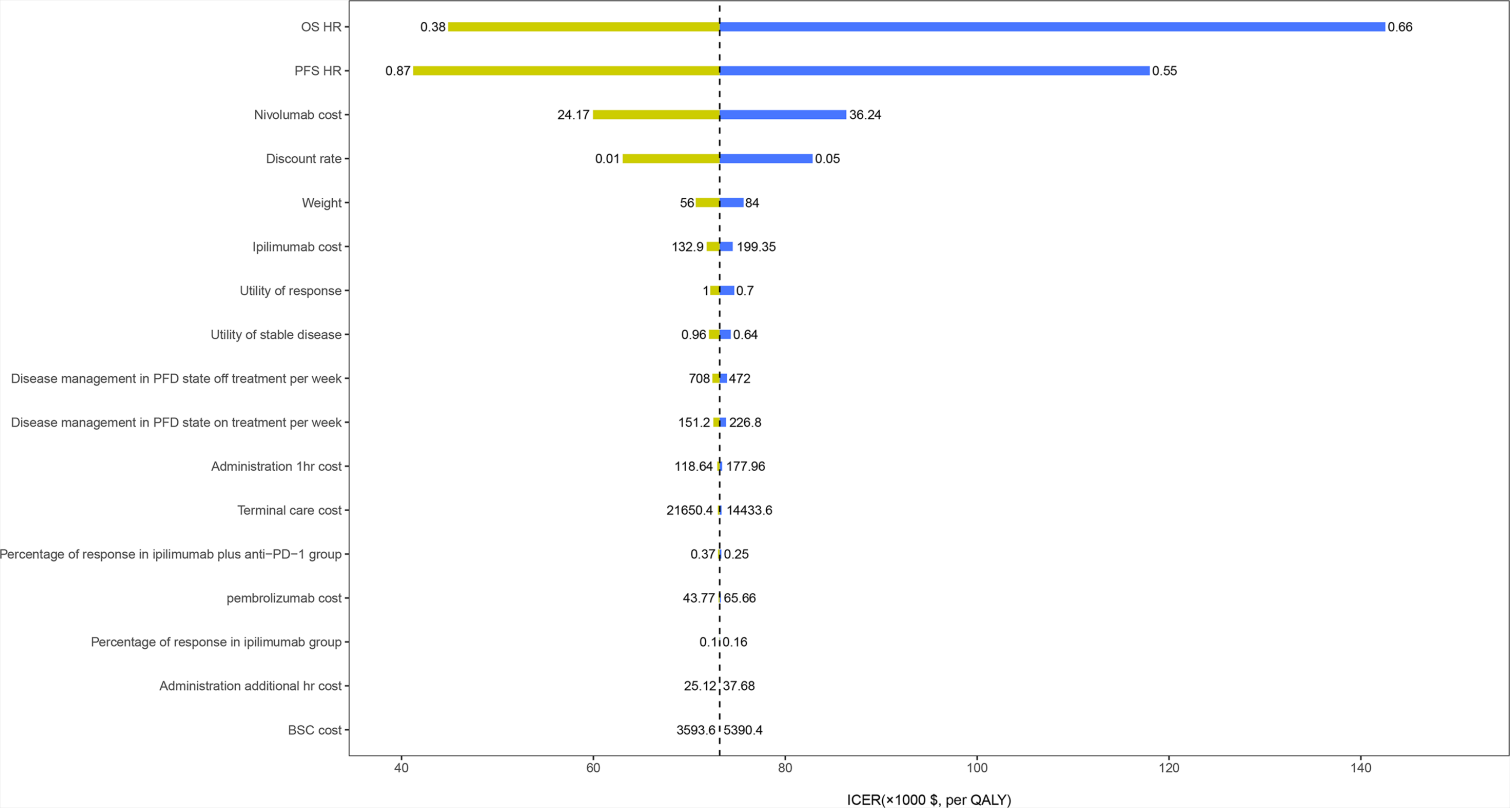
One-way sensitivity analyses results of ipilimumab plus anti-PD-1 strategy *versus* ipilimumab strategy in patients with metastatic melanoma resistant to anti-PD-(L)1 monotherapy. OS, overall survival; PFS, progression-free survival; HR, hazard ratio; BSC, best supportive care; PFD, progression-free disease.

**Figure 3:**
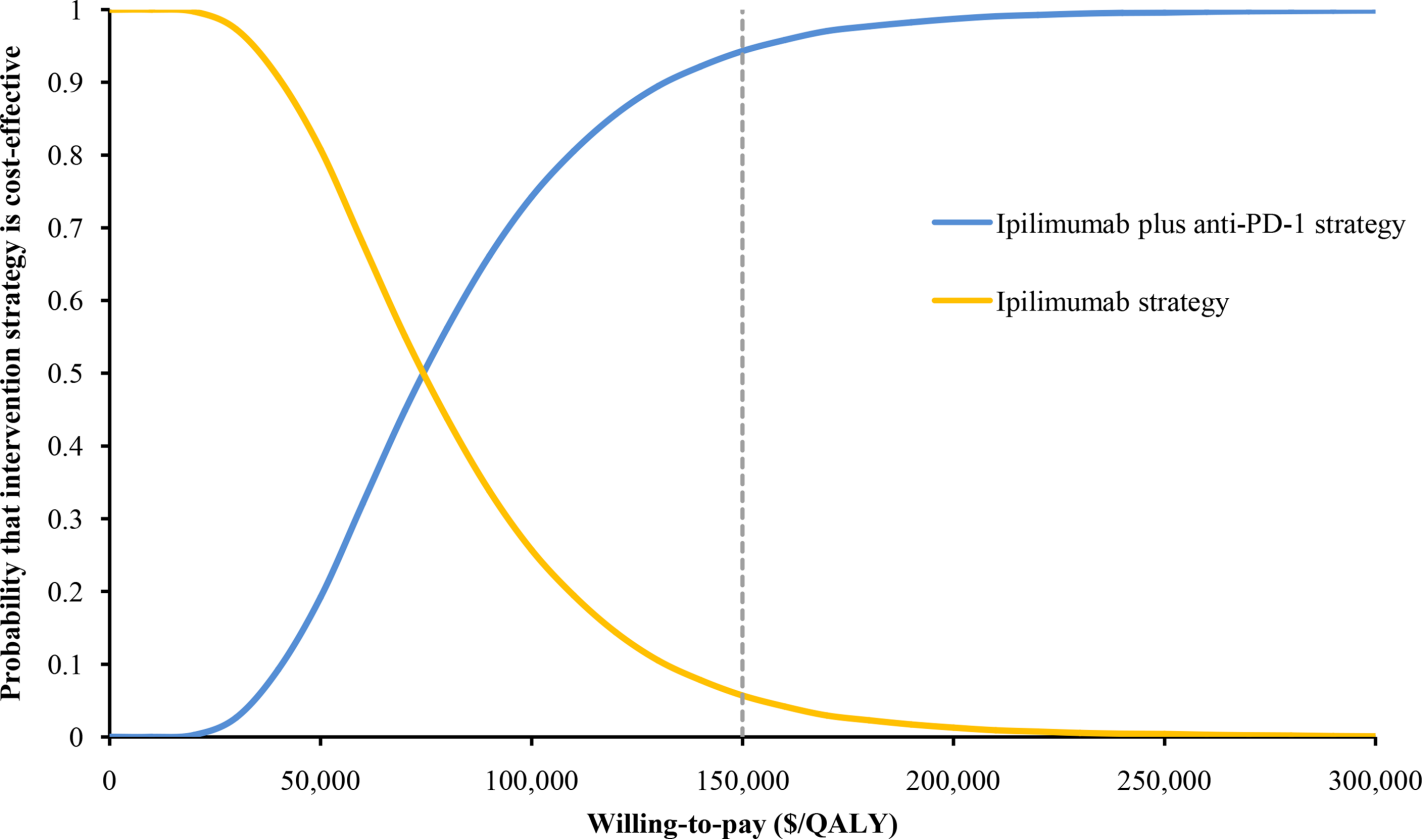
The cost-effectiveness acceptability curve of ipilimumab plus anti-PD-1 strategy and ipilimumab strategy in patients with metastatic melanoma resistant to anti-PD-(L)1 monotherapy.

The HR of OS for ipilimumab plus anti-PD-1 relative to ipilimumab was the most sensitive parameter (**Figure 2**). Other parameters such as the PFS HR, the cost of nivolumab and ipilimumab, and the utility values had a moderate or small influence on the ICER. Of note, all ICERs were below the WTP threshold of $150,000/QALY regardless of how the parameters vary within the range. In the probabilistic sensitivity analyses, the probability of ipilimumab plus anti-PD-1 being cost-effective was 19%, 75%, and 94% at the WTP of $50,000/QALY, $100,000/QALY, and $150,000/QALY, respectively (**Figure 3**).

### Subgroup Analyses

Compared with ipilimumab, ipilimumab plus anti-PD-1therapy for patients with metastatic melanoma resistant to anti-PD-(L)1 monotherapy showed ICERs below the WTP threshold of $150,000/QALY in the majority of the subgroups (**Table 3**). By varying the HRs of PFS and OS between the ipilimumab plus anti-PD-1 and ipilimumab strategies, the ICER of the subgroups varied from $39,433/QALY (probabilities of cost-effectiveness, 90%) in patients with systemic treatment between PD-(L)1 and ipilimumab plus anti-PD-1 to $388,947/QALY (probabilities of cost-effectiveness, 39%) in patients with anti-PD-1 adjuvant therapy. (**Table 3**).

**Table 3:** Results for subgroup analyses.

Subgroup	PFS HR (95% CI)	OS HR (95% CI)	ICER ($/QALY)	Cost-effectivenes probability at the threshold $150,000/QALY
Male	0·69 (0·51, 0·92)	0·45 (0·31, 0·64)	59,674	94%
Female	0·70 (0·48, 1·01)	0·60 (0·39, 0·92)	107,140	69%
Age<64 yr	0·72 (0·52, 1·00)	0·54 (0·37, 0·80)	78,301	84%
Age ≥ 64 yr	0·71 (0·51, 0·98)	0·48 (0·32, 0·73)	63,118	92%
BRAF mutant	1·01 (0·66, 1·56)	0·73 (0·43, 1·26)	80030	68%
NRAS mutant	0·62 (0·36, 1·07)	0·48 (0·25, 0·91)	85,476	74%
BRAF & NRAS WT	0·58 (0·42, 0·81)	0·46 (0·31, 0·69)	90,992	80%
ECOG PS 0	0·68 (0·49, 0·95)	0·47 (0·31, 0·72)	66,942	91%
ECOG PS ≥1	0·99 (0·70, 1·42)	0·82 (0·56, 1·21)	148,757	51%
III/M1A/M1B	0·74 (0·48, 1·15)	0·53 (0·30, 0·93)	70,480	80%
M1C/M1D	0·68 (0·51, 0·89)	0·50 (0·37, 0·69)	75,616	87%
Brain metastases				
No	0·79 (0·52, 1·18)	0·51 (0·31, 0·82)	55,313	87%
Yes	0·64 (0·48, 0·84)	0·47 (0·33, 0·66)	76,679	87%
Lung metastases				
No	0·73 (0·54, 0·98)	0·54 (0·38, 0·78)	75,857	85%
Yes	0·69 (0·48, 1·00)	0·48 (0·31, 0·74)	67,431	90%
Liver metastases				
No	0·68 (0·45, 1·04)	0·59 (0·37, 0·93)	109,323	64%
Yes	0·71 (0·54, 0·94)	0·48 (0·34, 0·67)	63,118	92%
LDH = UNL	0·75 (0·54, 1·02)	0·45 (0·30, 0·69)	48,906	97%
LDH >UNL	0·56 (0·38, 0·83)	0·47 (0·30, 0·72)	101,603	74%
PD-(L)1 treatment setting				
Adjuvant	0·52 (0·22, 1·25)	0·81 (0·17, 3·90)	388,947	39%
Metastatic	0·74 (0·58, 0·94)	0·54 (0·40, 0·71)	73,498	86%
Type of resistance with PD-(L)1				
Innate	0·75 (0·56, 0·99)	0·52 (0·37, 0·71)	65,477	90%
Acquired	0·71 (0·44, 1·14)	0·56 (0·31, 1·01)	87,840	73%
Best objective response with PD-(L)1				
SD/PD	0·72 (0·55, 0·95)	0·50 (0·36, 0·68)	66,335	90%
CR/PR	0·79 (0·45, 1·40)	0·70 (0·34, 1·43)	128,713	56%
Not applicable	0·52 (0·22, 1·25)	0·81 (0·17, 3·90)	388,947	39%
Time to recurrence/progression with PD-(L)1				
≤3 months	0·63 (0·46, 0.87)	0·45 (0·31, 0·64)	73,304	89%
>3 months	0·75 (0·53, 1·05)	0·52 (0·34, 0·81)	65,477	91%
Time from PD-(L)1 to IPI+/-PD1				
≤1 month	0·72 (0·52, 0·99)	0·54 (0·37, 0·80)	78,301	85%
>1 month	0·69 (0·49, 0·96)	0·47 (0·31, 0·70)	64,739	92%
Systemic treatment between PD-(L)1 and IPI+/-PD1				
No	0·67 (0·52, 0·87)	0·51 (0·38, 0·69)	81,393	84%
Yes	0·68 (0·36, 1·30)	0.34 (0·16, 0·75)	39,433	90%

OS, overall survival; PFS, progression-free survival; HR, hazard ratio; QALYs, quality-adjusted life-years; ICER, incremental cost-effectiveness ratio; LDH, lactate dehydrogenase; UNL, upper normal limit; WT, wild type; ECOG PS, Eastern Cooperative Oncology Group Performance Status; SD, stable disease; PD, progressed disease; CR, complete response; PR, partial response.

## Discussion

The study of Pires da Silva et al. demonstrated that ipilimumab plus anti-PD-1 can significantly improve PFS and OS in patients with metastatic melanoma who are resistant to anti-PD-(L)1 (4). However, since anti-PD-1 is a high-cost treatment, long-term administration before the disease progression may result in substantial cumulative medical expenditure. Our model suggests that ipilimumab plus anti-PD-1 may be a cost-effective option for patients with metastatic melanoma who are resistant to anti-PD-(L)1 compared to ipilimumab, with an ICER of $73,163/QALY. And ICERs in the majority of the subgroups were below the WTP threshold of $150,000/QALY.

In recent years, the emergence of novel anti-cancer therapies has not only improved the survival of patients but also increased the financial burden of patients (13). This situation is particularly serious in high-income countries, and the total expenditure on novel anti-cancer drugs in these countries has continued to increase (13). Therefore, it is becoming more and more important to provide safe healthcare in a cost-effective manner. At the clinical level, the economic evaluation of drugs can help physicians and patients evaluate the value of new cancer therapy in comparison with other prevailing clinical standard care. At the societal level, this type of analysis can help the government and pharmaceutical benefit providers to effectively and efficiently use limited medical resources (14). These will contribute to the sustainable development of the health care system that provides high-value care to all patients. The nature of ipilimumab plus anti-PD-1 to prolong the survival of metastatic melanoma was the main factor affecting economic outcomes. This was consistent with the results of our one-way sensitivity analysis, which showed that the HR of OS was the most sensitive parameter. Therefore, ipilimumab plus anti-PD-1 would have a higher probability of being cost-effective in the subgroup of patients with more favorable HR of OS, such as patients with upper limits of normal for lactate dehydrogenase, male and age ≥64 years. However, there was a low probability of ipilimumab plus anti-PD-1 therapy being cost-effective in patients with unfavorable HR of OS, such as patients with anti-PD-1 adjuvant therapy.

To our knowledge, this study was the first to evaluate the cost-effectiveness analysis of immune checkpoint inhibition in the second-line treatment of metastatic melanoma from the perspective of the United States. Prior studies have evaluated the economic value of immune checkpoint inhibition in the first-Line treatment of metastatic melanoma (1, 9, 15, 16); although the ICERs reported in these studies vary, many indicate that ipilimumab combined with immune checkpoint inhibition is likely to be cost-effective compared to ipilimumab monotherapy. For instance, Oh A et al (1) used data from the CheckMate-067 trial to evaluate the economics of nivolumab, ipilimumab, and nivolumab plus ipilimumab in the frontline treatment of metastatic melanoma. The results showed that nivolumab plus ipilimumab was likely to be cost-effective compared with ipilimumab monotherapy. A study by Bin Wu et al (16) showed that nivolumab plus ipilimumab was a cost-effective option for the treatment of newly diagnosed advanced melanoma.

Several important limitations were included in this analysis. First, the retrospective analysis on which it is based has a relatively small sample size. Only 162 patients with ipilimumab monotherapy and 193 patients with ipilimumab plus anti-PD-1 were included, especially the small size of patients receiving ipilimumab plus pembrolizumab. This lead to the limited power of this analysis. However, there was limited evidence on the effect of ipilimumab plus anti-PD-1 in the second-line treatment of metastatic melanoma. There was no doubt that the results of this analysis should be validated in a larger patient population in the future if valuable data were available. Second, there is compelling anti-PD-1 monotherapy, such as nivolumab or pembrolizumab (17), which could be used as comparators in our model instead of only ipilimumab. However, there was currently no study on the direct comparison between anti-PD-1 combination therapy and anti-PD-1 monotherapy as second-line treatments for metastatic melanoma. And the assumptions in the indirect comparison method will weaken the validity of our model, so we did not analyze them. When new clinical data were available, we would update the current analysis promptly. Third, considering that there was no significant difference in the incidence of AEs between the two groups, the cost of AEs was not included in the model. This may result in an overestimation of economic results. Therefore, the conclusions of this study should be carefully interpreted and referenced. Fourth, Some costs, such as testing or hospitalization, were not incorporated because they were considered to be the same between each arm of the model and would not affect the calculation of the incremental C/E ratio (ICER). Therefore, even if these costs were included, the main results and conclusions would not be changed. Despite these limitations, it may be valuable to provide physicians and policy-makers with the present results of this current study on metastatic melanoma.

In conclusion, for patients with metastatic melanoma who are resistant to anti-PD-(L)1, our study suggested that ipilimumab plus anti-PD-1 was a more cost-effective strategy compared to ipilimumab. These findings might help clinicians make optimal decisions in the treatment of metastatic melanoma.

## Data Availability Statement

The original contributions presented in the study are included in the article/supplementary material. Further inquiries can be directed to the corresponding authors.

## Author Contributions

Study design and supervision: XW and CT. Data analysis and interpretation: SL, LY, and XL. Data collection: LP, LW, SQ, and QL. Manuscript writing: YP and XZ. All authors contributed to the article and approved the submitted version.

## Funding

The work was supported by grants from the National Natural Science Foundation of China (grant numbers: 82073818 and 71874209); and the Key Science-Technology Research and Development Program of Hunan Province (grant number: 2020JJ8046); and the Hunan Provincial Natural Science Foundation of China (grant number: 2019JJ40411). The Rapid Service Fee was funded by the study sponsor.

## Conflict of Interest

The authors declare that the research was conducted in the absence of any commercial or financial relationships that could be construed as a potential conflict of interest.

## Publisher’s Note

All claims expressed in this article are solely those of the authors and do not necessarily represent those of their affiliated organizations, or those of the publisher, the editors and the reviewers. Any product that may be evaluated in this article, or claim that may be made by its manufacturer, is not guaranteed or endorsed by the publisher.
